# Supplementary material for: Tumor-Associated Neutrophils Dampen Adaptive Immunity and Promote Cutaneous Squamous Cell Carcinoma Development
Source: Cancers (Basel). 2020 Jul 10;12(7):1860. doi: 10.3390/cancers12071860 (PMC7408986; doi:10.3390/cancers12071860)
Supplement: Supplementary file 1 [file cancers-12-01860-s001.pdf]

cancers

*Article*

# Tumor-associated neutrophils dampen adaptive immunity and promote cutaneous squamous cell carcinoma development

Sokchea Khou, Alexandra Popa, Carmelo Luci, Franck Bihl, Aida Meghraoui-Kheddar, Pierre Bourdely, Emie Salavagione, Estelle Cosson, Alain Rubod, Julie Cazareth, Pascal Barbry, Bernard Mari, Roger Rezzonico, Fabienne Anjuère, Veronique M. Braud

## Supplementary Materials

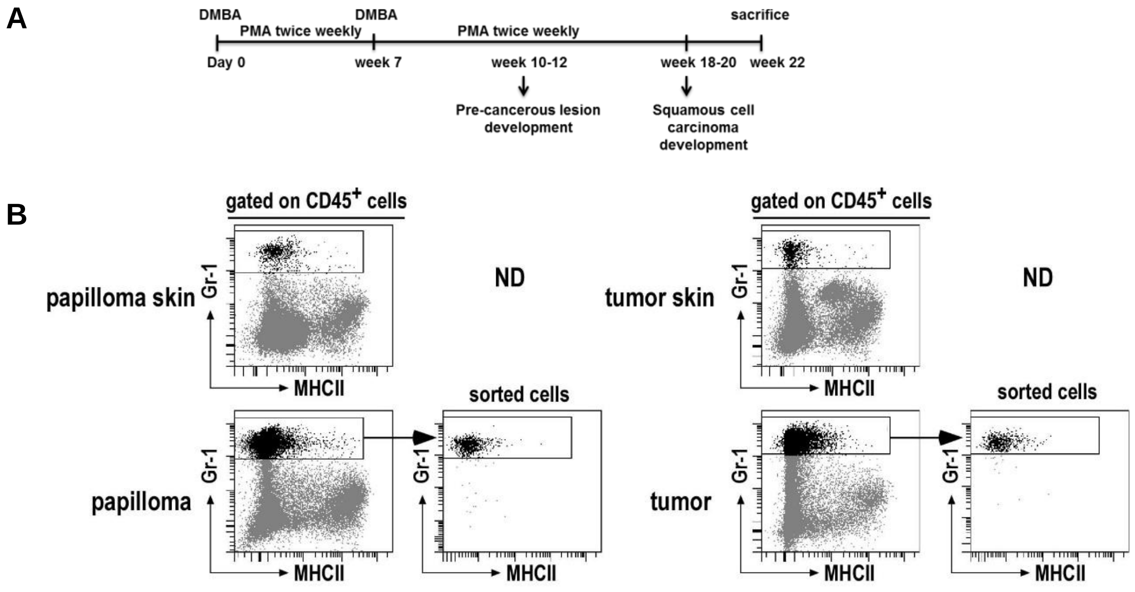

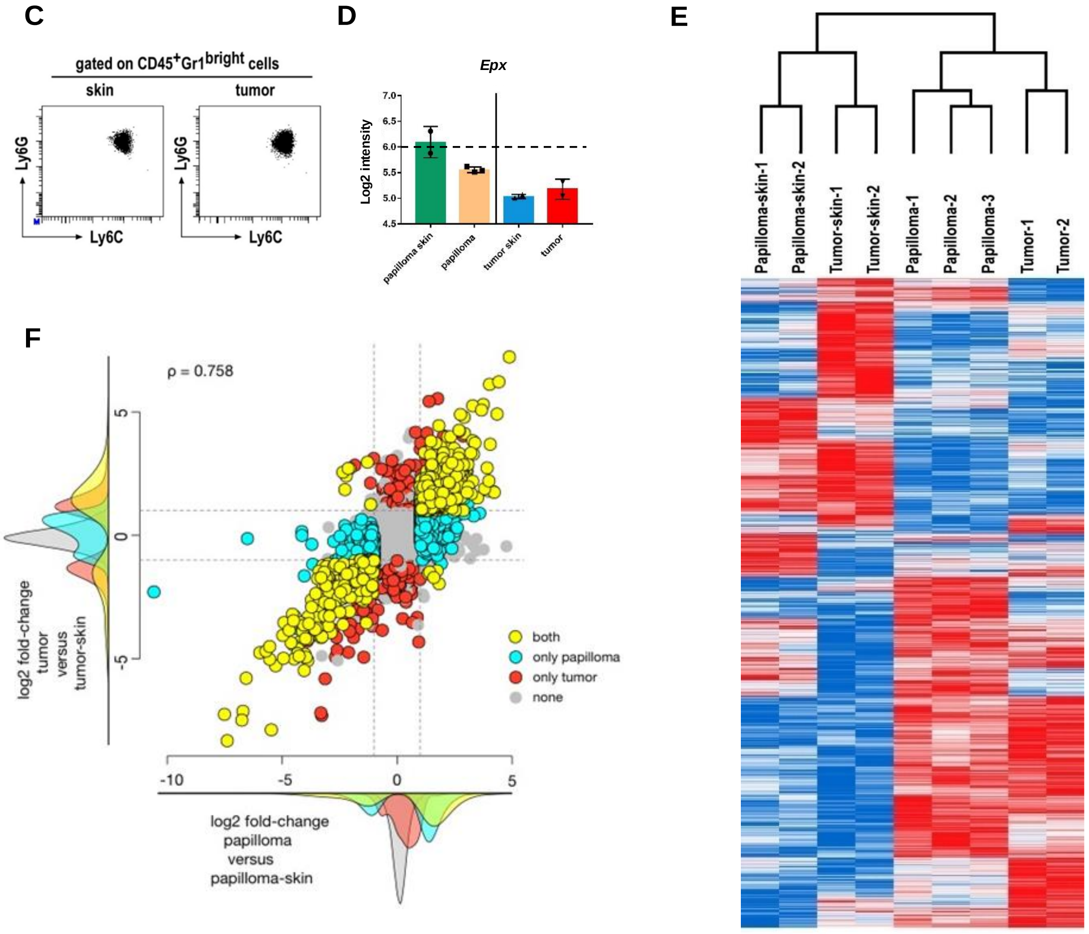


**Figure S1.** Gene-expression analyses of cell-sorted neutrophils purified from skin and chemically-induced cSCC, related to Figure 1. (**A**) Multi-stage chemical carcinogenesis. DMBA/PMA treatment protocol of FVB/N mice induces pre-cancerous lesions (papillomas) ~weeks 10–12, some further converting into squamous cell carcinomas (tumor) ~weeks 18–20. (**B**) Multiparameter flow cytometry cell sorting of $Gr\text{-}1^{bright}$ cells. Representative dot plots of $CD45^{+}$ pre-gated cells before and after $Gr\text{-}1^{bright}$-sorted cells (black gate), ND: not determined. (**C**) $Gr\text{-}1^{bright}$ cells identify $Ly6G^{+}$ neutrophils. Representative flow cytometry dot plots of $CD45^{+}Gr\text{-}1^{bright}$ gated cells. (**D**) Average intensity expression levels of *Epx* transcripts coding for eosinophil peroxidase (EPO) are shown. Dotted line delineates threshold for expression set at log2 gene expression above 6. (**E**) Hierarchical clustering. The 15% most variable probe sets with a minimum log2 average expression of 6 were analyzed within the indicated purified $Gr\text{-}1^{bright}$ cell populations (numbers relate to experiments-see Figure 1B). Probe sets were median-centered and a clustering per rows and columns was performed with the average-linkage method. (**F**) GENAS biological correlation between tumor and papilloma modulation on all probe sets.

**A**

| Canonical pathways | -Log10 (*P* value) | | | Activation z-score | | |
|---|---|---|---|---|---|---|
| | papilloma versus papilloma skin | tumor versus tumor skin | tumor versus papilloma | papilloma versus papilloma skin | tumor versus tumor skin | tumor versus papilloma |
| Cholecystokinin/Gastrin-mediated Signaling | 3.71 | 2.85 | 0.00 | 2.32 | 2.50 | NA |
| IL-8 Signaling | 8.31 | 5.52 | 1.97 | 2.92 | 1.26 | -2.24 |
| HMGB1 Signaling | 3.87 | 2.46 | 2.87 | 1.21 | 1.21 | -2.45 |
| ILK Signaling | 5.47 | 3.36 | 4.72 | 1.88 | -0.41 | -2.65 |
| Integrin Signaling | 5.81 | 2.53 | 3.69 | 1.63 | -1.46 | -3.00 |
| Signaling by Rho Family GTPases | 4.97 | 4.94 | 3.88 | 1.22 | -2.69 | -2.12 |
| RhoA Signaling | 1.01 | 2.38 | 2.11 | 1.00 | -2.00 | -2.00 |
| Agrin Interactions at Neuromuscular Junction | 2.58 | 3.67 | 2.29 | 0.71 | -1.15 | -2.00 |
| Cardiac Hypertrophy Signaling | 3.67 | 1.47 | 2.72 | 0.63 | -1.28 | -2.65 |
| Regulation of Actin-based Motility by Rho | 2.63 | 3.36 | 2.65 | 0.58 | -2.32 | -2.24 |
| Tec Kinase Signaling | 2.89 | 1.91 | 1.66 | -0.28 | -2.00 | NA |
| Rac Signaling | 0.54 | 1.93 | Inf | -0.38 | -2.67 | 0.00 |
| Role of NFAT in Cardiac Hypertrophy | 1.96 | 0.77 | 2.02 | -0.50 | -1.00 | -2.24 |
| HGF Signaling | 1.72 | 3.56 | 0.00 | -0.63 | -2.32 | NA |
| Leukocyte Extravasation Signaling | 3.25 | 3.30 | 1.30 | -0.77 | -2.40 | -2.24 |
| LXR/RXR Activation | 3.33 | 2.42 | 1.47 | -1.73 | -2.84 | 0.00 |
| PPAR Signaling | 4.66 | 3.20 | 0.21 | -2.32 | -2.50 | NA |
| Cell Cycle: G1/S Checkpoint Regulation | 0.64 | 1.52 | 0.87 | NA | 2.12 | NA |
| RhoGDI Signaling | 4.56 | 3.48 | 3.43 | -1.41 | 0.85 | 2.45 |

**B**

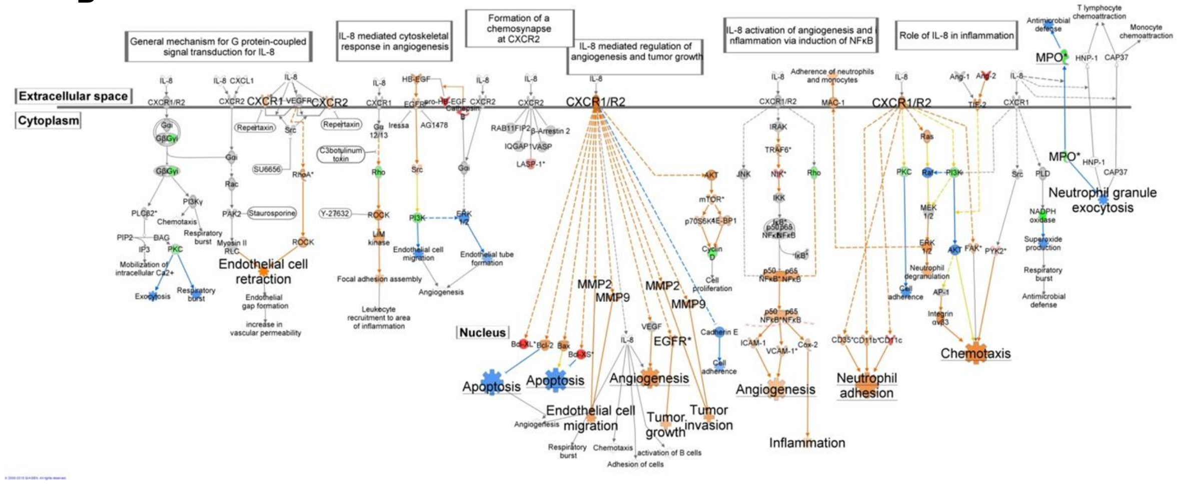


**C**

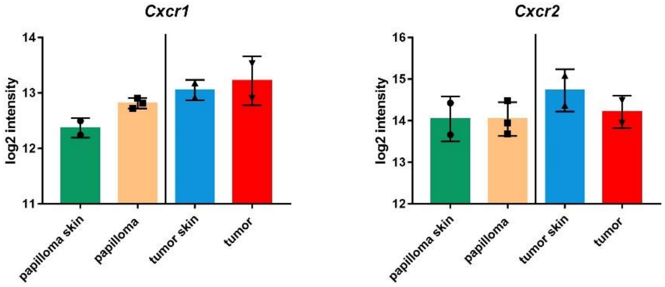


**Figure S2.** IPA Canonical Pathway enrichments, related to Figure 1. (**A**) Analysis of DEGs in papilloma and tumor versus their respective skin controls and their pairwise comparison. The most significant (-Log10 *p*-value ≥ 1.39) canonical pathways are shown. A negative z-score (blue) denotes an inhibited pathway. A positive z-score (orange) stands for an activated pathway. (**B**) IL-8 Signaling Canonical Pathway. Overlay of DEGs from papilloma versus papilloma skin. Red and green colors stand for genes up and down-regulated respectively. A molecular activity predictor (MAP) was superimposed. Orange and blue stand for activation and inhibition of the gene or function. (**C**) Average intensity expression levels of *Cxcr1 and Cxcr2* transcripts are shown.

| | PantherPathways | | | Biological Process | | | Protein Class | | | Cellular Component | | |
|---|---|---|---|---|---|---|---|---|---|---|---|---|
| | Class | Fold enrichment | -log10 (P-value) | Class | Fold enrichment | -log10 (P-value) | Class | Fold enrichment | -log10 (P-value) | Class | Fold enrichment | -log10 (P-value) |
| **Cluster 1** | | | | endocytosis | > 5 | 1,50 | extracellular matrix structural protein | > 5 | 1,57 | | | |
| **Cluster 2** | Cytoskeletal regulation by Rho GTPase | > 5 | 1,82 | cellular component organization | 2,99 | 1,96 | | | | | | |
| | | | | cellular process | 1,57 | 1,76 | | | | | | |
| | | | | cellular component morphogenesis | 4,48 | 1,71 | | | | | | |
| | | | | anatomical structure morphogenesis | 3,93 | 1,57 | | | | | | |
| | | | | cellular component organization or biogenesis | 2,73 | 1,48 | | | | | | |
| **Cluster3** | Heterotrimeric G-protein signaling pathway-Gq alpha and Go alpha mediated pathway | > 5 | 3,39 | regulation of molecular function | 2,29 | 2,93 | G-protein modulator | 3,73 | 4,41 | actin cytoskeleton | 3,07 | 1,93 |
| | Inflammation mediated by chemokine and cytokine signaling pathway | 3,69 | 1,69 | regulation of catalytic activity | 2,27 | 2,71 | guanyl-nucleotide exchange factor | 4,61 | 2,13 | | | |
| | Pentose phosphate pathway | > 5 | 1,40 | metabolic process | 1,33 | 2,68 | enzyme modulator | 1,97 | 1,84 | | | |
| | B cell activation | > 5 | 1,39 | | | | | | | | | |
| **Cluster 4** | | | | mesoderm development | 4,69 | 5,25 | receptor | 2,46 | 2,33 | | | |
| | | | | immune system process | 3,06 | 4,20 | defense/immunity protein | 3,75 | 2,00 | | | |
| | | | | response to stimulus | 2,38 | 3,82 | cell adhesion molecule | 4,14 | 1,79 | | | |
| | | | | angiogenesis | > 5 | 2,99 | | | | | | |
| | | | | cell communication | 1,98 | 2,13 | | | | | | |
| | | | | system development | 2,74 | 2,05 | | | | | | |
| | | | | developmental process | 2,12 | 1,94 | | | | | | |
| **Cluster 5** | | | | | | | | | | | | |
| **Cluster 6** | | | | | | | | | | | | |
| **Cluster 7** | Integrin signalling pathway | > 5 | 8,40 | cell communication | 2,52 | 10,18 | extracellular matrix protein | > 5 | 11,69 | extracellular region | > 5 | 15,08 |
| | | | | biological adhesion | > 5 | 10,17 | signaling molecule | 3,64 | 7,28 | extracellular matrix | > 5 | 14,76 |
| | | | | cell-cell adhesion | > 5 | 9,15 | extracellular matrix structural protein | > 5 | 5,61 | | | |
| | | | | cell adhesion | > 5 | 8,99 | extracellular matrix linker protein | > 5 | 4,72 | | | |
| | | | | developmental process | 2,65 | 8,36 | surfactant | > 5 | 4,06 | | | |
| | | | | cellular process | 1,75 | 8,10 | cell adhesion molecule | 4,26 | 3,52 | | | |
| | | | | ectoderm development | 4,37 | 5,69 | receptor | 2,3 | 2,91 | | | |
| | | | | system process | 2,8 | 4,98 | membrane-bound signaling molecule | > 5 | 1,83 | | | |
| | | | | single-multicellular organism process | 2,56 | 4,83 | extracellular matrix glycoprotein | > 5 | 1,79 | | | |
| | | | | multicellular organismal process | 2,56 | 4,81 | nucleic acid binding | 0,26 | 1,78 | | | |
| | | | | regulation of liquid surface tension | > 5 | 4,03 | antibacterial response protein | > 5 | 1,43 | | | |
| | | | | cell-matrix adhesion | > 5 | 3,76 | | | | | | |
| | | | | immune system process | 2,58 | 3,60 | | | | | | |
| | | | | homeostatic process | > 5 | 3,52 | | | | | | |
| | | | | mesoderm development | 3,49 | 3,48 | | | | | | |
| | | | | system development | 2,71 | 3,45 | | | | | | |
| | | | | cell-cell signaling | 3,51 | 2,70 | | | | | | |
| | | | | macrophage activation | > 5 | 2,50 | | | | | | |
| | | | | nervous system development | 3 | 2,29 | | | | | | |
| | | | | endocytosis | 3,89 | 1,79 | | | | | | |
| | | | | response to stimulus | 1,88 | 1,77 | | | | | | |
| | | | | receptor-mediated endocytosis | > 5 | 1,74 | | | | | | |
| **Cluster 8** | Glycolysis | > 5 | 1,40 | glycolysis | > 5 | 2,61 | | | | intermediate filament cytoskeleton | > 5 | 1,35 |
| **Cluster 9** | Glycolysis | > 5 | 2,47 | glycolysis | > 5 | 2,40 | cysteine protease inhibitor | > 5 | 2,48 | | | |
| | | | | monosaccharide metabolic process | > 5 | 1,57 | | | | | | |
| **Cluster 10** | CCKR signaling map | > 5 | 2,09 | cellular component biogenesis | 4,2 | 2,39 | | | | | | |
| | | | | metabolic process | 1,32 | 1,57 | | | | | | |
| | | | | nitrogen compound metabolic process | 2,26 | 1,51 | | | | | | |
| | | | | cellular component organization or biogenesis | 2,08 | 1,30 | | | | | | |

**Figure 3.** Gene cluster GO-enrichment analyses, related to Figure 1. PantherDB (http://pantherdb.org/).

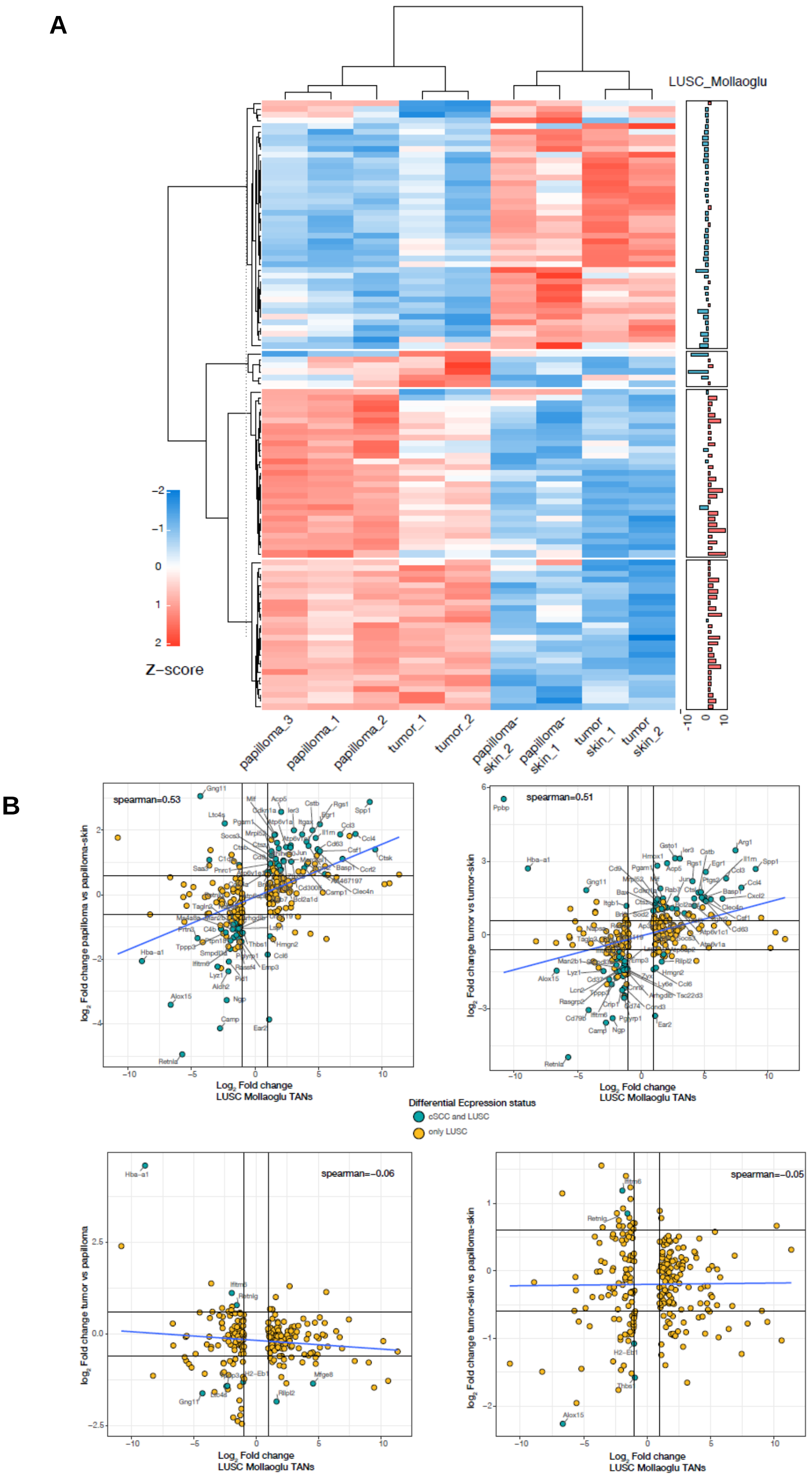


**Figure S4.** Common transcriptomic modulation signature between TANs in two different types of SCC, from the skin or lung, related to Figure 1. (**A**) Hierarchical clustering of differentially expressed genes from neutrophils in cSCC compared to skin control (see Figure 1) and in lung SCC compared to circulating neutrophils [35]. Only genes differentially expressed in both studies (MaxExp ≥ 5, adjusted *p*-value ≤ 0.05 and absolute log2 fold-change ≥ 0.6) were considered. The log2 fold-change of each gene in the Lung SCC model between TAN and circulating neutrophils is reported as barplots on the right side of the figure. Red color of the barplot means up-regulation in the TANs, while blue stands for down-regulation. (**B**) Pairwise comparisons of the log2 fold-change modulation between [35] and our study for all genes reported as modulated in [35]. The spearman coefficient is reported for each comparison. Genes that are significantly modulated in both datasets are reported in blue, while those modulated only in the dataset [35] are reported in yellow.

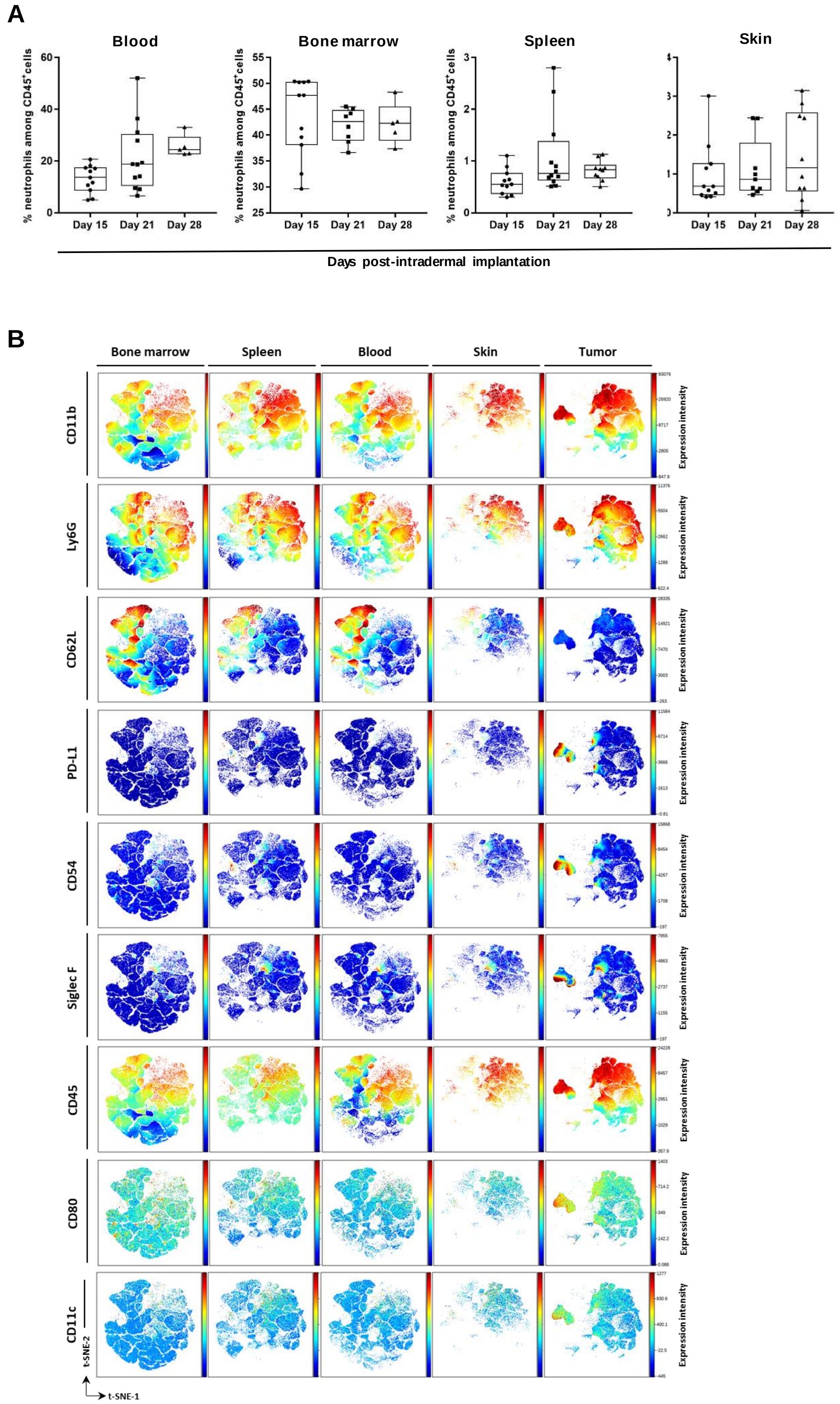


**Figure S5.** Neutrophil frequencies and phenotypes in mSCC38-bearing mice, related to Figure 2. (**A**) Frequencies of neutrophils overtime in the indicated tissue compartments of mSCC38-bearing mice ($n$ = 5–11, mean ± SEM from three independent experiments). (**B**) t-SNE analysis was performed on pre-gated CD45$^+$Ly6G$^+$ neutrophils from all samples. Neutrophils markers expression is presented on a rainbow heat scale in the t-SNE map of each group concatenated file.

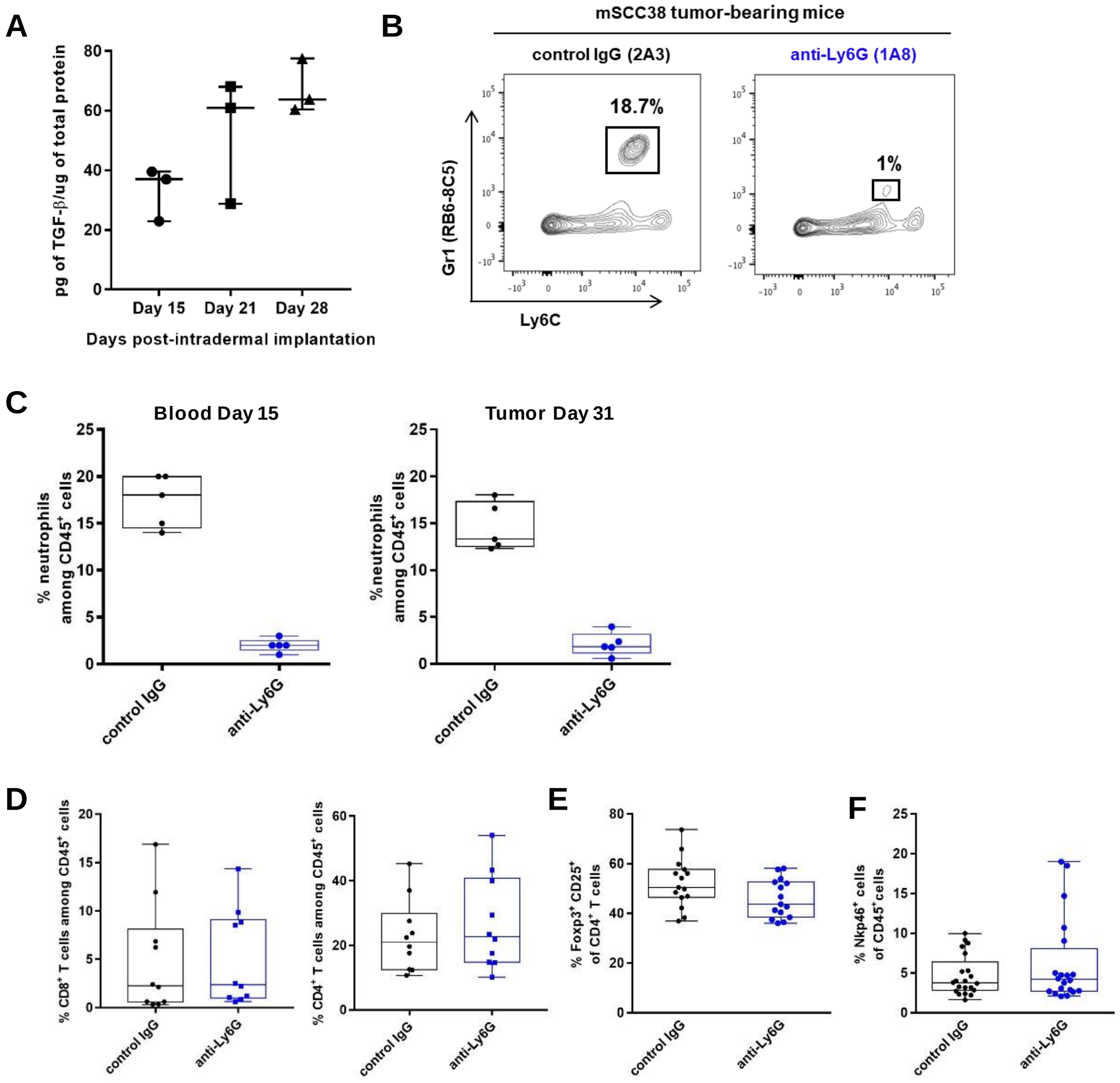


**Figure S6.** Analyses of tumor microenvironment of WT and neutrophil-depleted mice, related to Figure 3. (**A**) TGF-β concentration in mSCC38 tumors ($n$ = 3, mean ± SD). (**B**) Representative contour plot showing expression of Gr1 and Ly6C in CD45+ cells from blood of isotype IgG control or anti-Ly6G-treated mice day 15 post-intradermal implantation. (**C**) Frequencies of neutrophils monitored in blood and tumors of isotype IgG control or anti-Ly6G-treated mice. ($n$ = 5 mice, mean ± SD). (**D**) Frequencies of CD8+ and CD4+ T cells among CD45+ cells in mSCC38 tumors from isotype IgG control or anti-Ly6G-treated mice day 30 post-intradermal implantation, ($n$ = 10, mean ± SEM from two independent experiments). (**E**) Frequencies of FoxP3+ CD25+ Treg among CD4+ T cells in mSCC38 tumors from isotype IgG control or anti-Ly6G-treated mice day 30, ($n$ = 15, mean ± SEM from three independent experiments). $p$ value ns, Mann-Whitney $U$ test. (**F**) Frequencies of CD3− NKp46+ NK cells among CD45+ cells in mSCC38 tumors from isotype IgG control or anti-Ly6G-treated mice day 30, ($n$ = 20, mean ± SEM from three independent experiments).

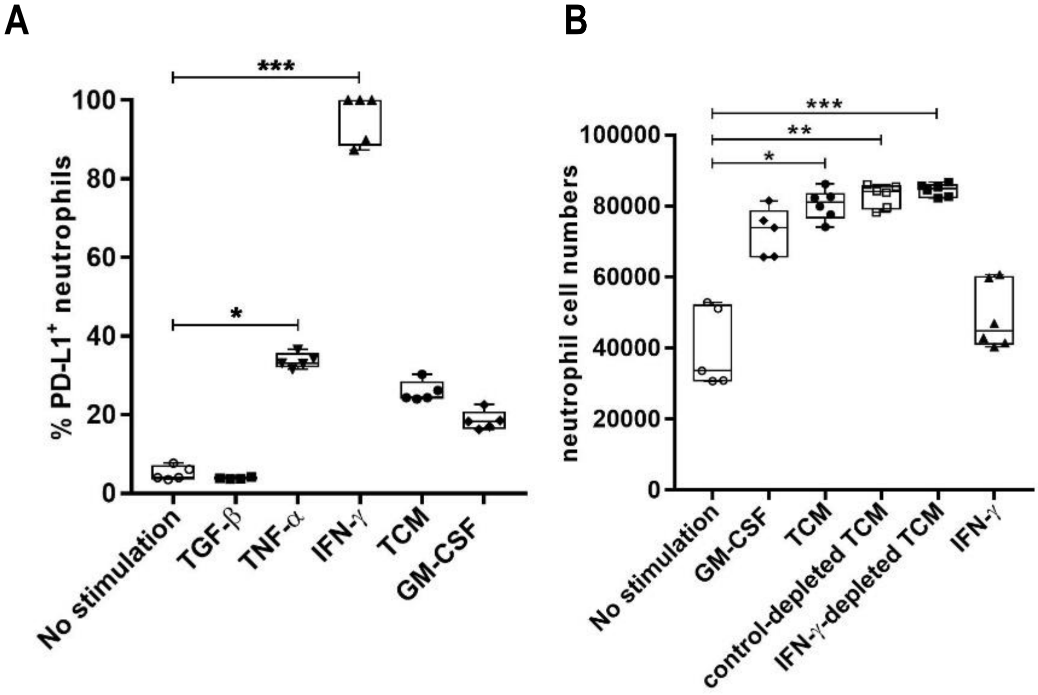


**Figure S7.** Modulation of PD-L1 cell surface expression and survival of neutrophils, related to Figure 4. (**A**) Frequencies of PD-L1⁺ neutrophils purified from BM and stimulated in vitro for 24 h with medium alone or in the presence of 500 ng/mL TGF-β, 500 ng/mL TNF-$\alpha$, 10ng/ml IFN-$\gamma$, TCM (1/2) or 500 ng/mL GM-CSF. *$p < 0.05$, ***$p < 0.001$, Kruskal-Wallis one-way ANOVA. (**B**) neutrophil cell numbers upon culture 24 h in media alone, or supplemented with indicated TCM or cytokines. *$p < 0.05$, **$p < 0.01$, Kruskal-Wallis one-way ANOVA.

© 2020 by the authors. Licensee MDPI, Basel, Switzerland. This article is an open access article distributed under the terms and conditions of the Creative Commons Attribution (CC BY) license (http://creativecommons.org/licenses/by/4.0/).
